# Antispasmodic Activity of Prenylated Phenolic Compounds from the Root Bark of *Morus nigra*

**DOI:** 10.3390/molecules24132497

**Published:** 2019-07-08

**Authors:** Zoofishan Zoofishan, Norbert Kúsz, Attila Csorba, Gábor Tóth, Judit Hajagos-Tóth, Anna Kothencz, Róbert Gáspár, Attila Hunyadi

**Affiliations:** 1 Institute of Pharmacognosy, Interdisciplinary Excellence Center, University of Szeged, H-6720 Szeged, Hungary; 2 NMR Group, Department of Inorganic and Analytical Chemistry, Budapest University of Technology and Economics, Szt. Gellért Sq. 4, H-1111 Budapest, Hungary; 3 Department of Pharmacodynamics and Biopharmacy, University of Szeged, H-6720 Szeged, Hungary; 4 Department of Pharmacology and Pharmacotherapy, Interdisciplinary Excellence Centre, University of Szeged, H-670 Szeged, Hungary; 5 Interdisciplinary Centre for Natural Products, University of Szeged, H-6720 Szeged, Hungary

**Keywords:** mulberry polyphenol, prenylflavonoid, arylbenzofuran, gastrointestinal disorder, asthma, spasmolytic

## Abstract

Black mulberry is a widely acknowledged ancient traditional medicine. Its extract and constituents have been reported to exert various bioactivities including antimicrobial, hypotensive, analgesic etc. effects. While black mulberry preparations are also used as antispasmodic agents in folk medicine, no related studies are available on its isolated constituents. Through an extensive chromatographic purification, seven phenolic compounds were isolated from the methanol extract of *Morus nigra* root bark, including morusin (**1**), kuwanon U (**2**), kuwanon E (**3**), moracin P (**4**), moracin O (**5**), albanol A (**6**), and albanol B (**7**). A complete NMR signal assignment of moracin P and O was achieved, and related literature errors confusing the identity of moracin derivatives are hereby clarified. Compounds **2**, **5** and **7** were identified as strong antispasmodic agents on isolated rat ileum and tracheal smooth muscles, while compound **3**, a methoxy derivative of **2**, was inactive. Moracin O (**5**) inhibited the ileal and tracheal smooth muscle contractions with E_max_ values of 85% and 302 mg, respectively. Those actions were superior as compared with papaverine. Our findings demonstrate that prenylated arylbenzofurans, geranylated flavonoids and Diels-Alder adducts from *Morus nigra* are valuable antispasmodic agents. Compounds **2**, **5** and **7** are suggested as marker compounds for quality control of antispasmodic mulberry preparations. Moracin O (**5**) is a new lead compound for related drug development initiatives.

## 1. Introduction

Antispasmodics are widely used to treat conditions involving impaired contraction and relaxation of smooth muscles in order to relieve muscle spasms, breathing problems, gastrointestinal cramps, movement disorders, etc. [1]. Airway smooth muscles are responsible for the acute constriction of the trachea and bronchi in asthma [2]. Concerning gastrointestinal smooth muscle contractility, it represents the key mechanism involved in the pathophysiology of several gastrointestinal disorders including irritable bowel syndrome (IBS) [3]. Therefore, antispasmodics play an important role in the pharmacotherapy of such diseases. Considering the development of tolerance to present drugs and their potential adverse effects [4,5], the search for new antispasmodics that can act as bronchodilator agents and/or gastrointestinal smooth muscle relaxants are of great interest. In this context, the relaxant effect on airway and gastrointestinal smooth muscle has been reported for several medicinal plants, including the black mulberry [6,7].

*Morus nigra* L. (Moraceae), commonly known as the black mulberry, is cultivated worldwide for its nutritional, industrial and medicinal value. [8] According to a comparative study, *M. nigra* L. has the highest amount of total phenolic and flavonoid content as compared to other *Morus* species such as the white (*M. alba* L.) and the red (*M. rubra* L.) mulberry [9]. The root bark of *M. nigra* is a particularly rich source of prenylated phenolic compounds [10]. The medicinal properties of *M. nigra* have been valued since ancient times. In Greek and Ayurvedic medicines, preparations of this plant have been used in various disorders involving the gastrointestinal tract (dyspepsia, diarrhea and constipation) or the airways (sore throat, asthma and bronchitis), and also for the treatment of hypertension [11,12,13]. In India, the black mulberry plant is an active constituent for many Unani and ayurvedic medicines as mentioned in the ayurvedic pharmacopeia [14,15]. It can be used either as a single drug or a component of complex preparations to treat different disorders, such as for example “Tut-i-aswad”, an Unani medicine for cancer that contains the fruit of black mulberry [16]. “Rub-e-toot siyah”, an ayurvedic remedy made from the root bark, is widely used for sore throat or laryngitis [17]. Almost all parts of the plant are used in the folk medicine, including treatment of hepatic [18] and cardiovascular diseases [19], and as a diuretic [20], analgesic and hypoglycemic agent [21].

The aim of the present study was to investigate the possible smooth muscle relaxant activity of *M. nigra* root bark extract and its constituents through a multi-step chromatographic purification of the compounds and testing their antispasmodic effect on isolated rat trachea and ileum.

## 2. Results and Discussion

### 2.1. Isolation and Structure Elucidation of Compounds ***1***–***7***

**Figure 1:**
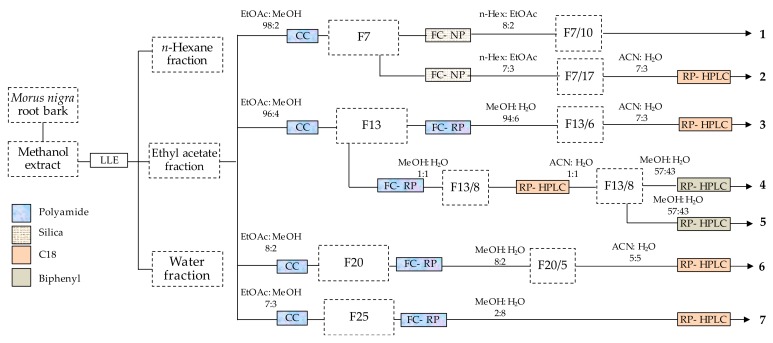
Procedure of isolating phenolic compounds from the root bark of *Morus nigra*. F numbers represent the corresponding fractions as detailed in the Materials and Methods, Section 3.3. LLE: liquid-liquid extraction), CC: column chromatography, FC-NP: flash chromatography on normal phase, FC-NP (flash chromatography on reverse phase, RP-HPLC: reverse-phase HPLC.

The root bark of *Morus nigra* is a rich source of bioactive phenolic compounds [10]. In this study, a multi-step chromatographic process was utilized for the isolation of such compounds. This process included a combination of various techniques with different selectivity. The purification was started by using polyamide column chromatography that is particularly efficient in separating phenolic compounds from those with alcoholic hydroxyl groups [22], and this was followed by normal- and/or reverse-phase flash chromatography, and reverse-phase HPLC. Seven phenolic compounds (**1**–**7**) were obtained as shown in Figure 1. 

Structure elucidation of compounds **1**–**3** and **6**–**7** was straightforward through comparing their high-resolution electrospray ionization mass spectrometry (HRESIMS) and/or ^1^H- and ^13^C-NMR data with published literature values [23,24,25,26,27]. However, when comparing the NMR chemical shifts of compounds **4** and **5** with literature data, we found some contradictory spectral data concerning moracin P and two related arylbenzofuran derivatives, moracins Q and R. In the paper reporting the first isolation of moracins Q and R [28], chemical shifts of the prenyl-originated dimethyldihydropyran ring of moracin Q, i.e., the dimethoxy derivative of moracin P, were assigned as nearly identical to those previously reported for the substituted dihydrofuran ring of moracin O [25]. Further, the same paper [28] reported chemical shifts of the prenyl side-chain of moracin R nearly identical to those previously published for the analogous prenyl-originated ring of moracin P [25]. 

First, we attempted to clarify this through HRESIMS, but both compounds **4** and **5** showed the same elemental compositions, i.e., C_19_H_18_O_5_. Considering that both moracins P and O may be formed from moracin R by a water elimination, this may still have meant that the molecular ion of moracin R could not be observed. It is worth noting that moracin R was reported with high-resolution mass spectral data referring to the [M − H_2_O]^+^ *m/z* value [28].

Compound **4** was obtained as a brown amorphous powder. The molecular formula was determined as C_19_H_18_O_5_ by HRESIMS. Signal assignment of the ^1^H-NMR spectrum (Figure S1) revealed the presence of a disubstituted 2-arylbenzofuran moiety, a trisubstituted 2,2-dimethyldihydropyran ring and a 1,3,5-trisubstituted benzene ring. The ^13^C APT (attached proton test) measurement (Figure S2) showed 16 signals corresponding to 18 carbon atoms, including two methyl, one methylene, one sp^3^ oxymethyne and one sp^3^ quaternary carbon, and in the sp^2^ region further six methyne, three quaternary carbon and five oxyaryl carbon atoms. The HSQC experiment (Figure S3) revealed the one-bond ^1^H/^13^C connections, while the HMBC measurement (Figure S4) allowed the assignment of the quaternary carbon atoms through the ^2^*J*_H,C_ and ^3^*J*_H,C_ couplings. Based on the above spectroscopic evidence, compound **4** was unambiguously identified as moracin P. Therefore, previous assignment of the structure of this compound was correct [25], and this conclusion is also supported by reports on other moracin P derivatives [29,30,31].

The condensed dimethyldihydropyran ring of moracin P (**4**) appeared in an equilibrium of two half-chair conformations where C-2″ and C.3″ were the atoms out of the plane. The unambiguous identification of the chemical shifts of the *cis* and *trans* located methyl/methine hydrogens was achieved utilizing the two-dimensional NOESY measurement (τ_mixing_ = 300 ms) (SP-5). The 2.83/1.28 cross-peak assigned that in the preferred conformation H-1″/CH_3_ (δ = 1.28) were *cis* and took the 1″/3″ diaxial position. The ^3^*J*(H_trans_-1″,H-2″) > ^3^*J*(H_cis_-1″, H-2″) values (7.6 Hz/5.2 Hz) supported the preference of the depicted conformation (Figure S5).

Compound **5** was obtained as a brown amorphous powder. The molecular formula was determined, also in this case, to be C_19_H_18_O_5_ by HRESIMS. The ^1^H (Figure S6) and ^13^C APT (Figure S7) NMR spectra exhibited rather similar signals as obtained for **4**, indicating that they should be structural isomers. The spectra suggested the presence of a disubstituted 2-arylbenzofuran moiety and a 1,3,5-trisubstituted benzene ring. Considering the heteronuclear multiple bond correlations (HMBC) of the δ H-2″ (4.64 ppm) atom with the δ C-5 (125.2 ppm) and δ C-6 (160.0 ppm), the third ring of compound **5** was a condensed dihydrofuran ring substituted with a 1-methyl-1-hydroxyethyl group (HO-C(Me_2_)-), suggesting that this compound should be moracin O. The NMR signal assignment was also supported by the heteronuclear single quantum coherence (HSQC) (Figure S8), HMBC (Figure S9) and NOESY (Figure S10) spectra. It needs to be stressed that NMR measurements taken in CD_3_OD, or in other solvents where no separated OH signals can be observed, provide no information on the number of the OH groups. To overcome this uncertainty, we have taken the NMR investigations (^1^H, ^13^C, edited HSQC, and HMBC, see Figures S11–S14) of compound **5** also in DMSO-*d_6_*.

A clear differentiation between moracin O and moracin R (**5a**) can be accomplished by detecting the OH signals in the ^1^H-NMR spectrum: moracin R contains five, whereas Moracin O only three OH groups. In the ^1^H spectrum, two new singlets appeared at δ 9.43 (2H) and 4.63 (1H), and these did not give HSQC cross-peaks (Figure S13), justifying the presence of three hydroxyl groups. Since the values of ^1^H and ^13^C chemical shifts slightly changed in DMSO-*d_6_* as compared to those observed in CD_3_OD, HSQC and HMBC experiments were also performed to establish a complete ^1^H and ^13^C signal assignment. The limited resolution in the F1 dimension (^13^C) of the routine HMBC experiment (optimized for *J_C,H_* = 8 Hz long-range couplings) did not allow the confident assignment of several quaternary ^13^C signals. To achieve the required extremely high ^13^C chemical shift resolution, the band-selective HMBC experiment was the method of our choice (see the upper part in Figure S14). The 9.43/158.8 ppm cross-peak assigned the 3ʹ,5ʹ-OH positions and the exact δ C-3ʹ,5ʹ chemical shifts, differentiating from the rather closely appearing C-6 (δ 158.3) peak. With the aid of the well separated HMBC cross-peaks 6.66/154.1 and 7.34/154.2 ppm, respectively, the unambiguous assignment of the δ C-2 and δ C-7a values became possible. Based on the above, compound **5** was clearly identified as moracin O. As for the contradictory literature data, this also suggests that the compound reported as moracin Q is likely the dimethoxy derivative of moracin O instead of that of moracin P. Further, the chemical shifts reported for moracin R suggest a possible false assignment of the structure of this compound that was moracin P instead [28].

**Figure 2:**
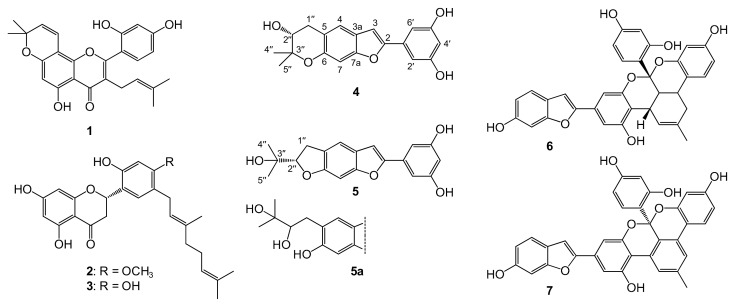
Phenolic compounds isolated from the root bark of *Morus nigra*. These are morusin (**1**), kuwanon U (**2**), kuwanon E (**3**), moracin P (**4**), moracin O (**5**), albanol A (**6**), and albanol B (**7**). Moracin R (**5a**) is presented only for comparison purposes as a ruled-out alternative structure for compound **5**; compound **5a** was not isolated in this study. Atomic numbering is presented for moracin P (**4**) and moracin O (**5**).

Compounds **4** and **7** are reported here for the first time from the root bark of *M. nigra*, whereas the presence of other compounds was previously reported [32,33]. Chemical structures of compounds **1**–**7**, as well as that of moracin R (**5a**), are presented in Figure 2.

### 2.2. Antispasmodic Activity of Compounds **1**–**7**

As a first screening, a 3-points assay was performed that was an appropriate experimental setup for the identification of active compounds, but it was not precise enough to determine the affinity (EC_50_) and efficacy (E_max_). In this preliminary bioassay, the crude extract and most of the solvent-solvent extracted fractions of *Morus nigra* root bark elicited a slight contracting effect (i.e., negative action) or no effect at all. These results suggest that the root bark of *Morus nigra* may contain constituents with smooth muscle contracting activity that mask the potent smooth muscle relaxing effect of some of the isolated compounds (see below). Morusin (**1**), kuwanon E (**3**), moracin P (**4**) and albanol A (**6**) had only non-significant relaxing activity (or no action) on the rat ileal contractions. Additionally, except for albanol A, these compounds elicited a very moderate tracheal tone reducing effect that was much lower than that of papaverine. However, a remarkable activity was found for kuwanon U (**2**), moracin O (**5**) and albanol B (**7**) on both experimental models (Table 1), therefore these compounds were further studied for their efficacy.

**Figure 3:**
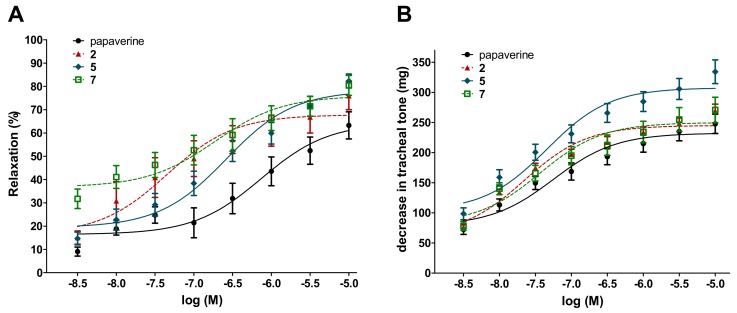
Concentration–response curves of compounds **2**, **5** and **7** on the ileal contractions (**A**) and tracheal tone (**B**) of isolated rat smooth muscles. Papaverine was used as a positive control. All the compounds elicited a relaxing effect on ileal smooth muscle.

Remarkably, the 8-points assay on the inhibition of ileal contractions revealed that compounds **2**, **5** and **7** were equipotent with papaverine with respect to their EC_50_ values in both experimental models. Furthermore, each of these compounds showed a tendency for a higher E_max_ value on ileal contraction than that of papaverine, and in the case of moracin O (**5**), this was also statistically significant. With respect to the compounds’ activity on the tracheal tone, similar results were obtained. The three studied compounds exerted their 50% activity at the same, low nanomolar concentration range as papaverine. Furthermore, moracin O (**5**) exerted a significantly stronger maximum decrease in the tracheal tone as compared to papaverine. Dose–response curves and calculated numerical results of these experiments are presented in Figure 3 and Table 2.

To the best of our knowledge, this is the first report of the smooth muscle relaxant activity of compounds **2**, **5**, and **7**. This bioactivity of each of these compounds is of high potential therapeutic interest: kuwanon U (**2**) and albanol B (**7**) were found equipotent with the opium alkaloid antispasmodic drug papaverine (i.e., no statistically significant differences were found between them), and moracin O (**5**) exerted an even stronger effect than that.

Vasorelaxant activity of several prenylated phenolic compounds has previously been reported, but much higher concentrations typically in the medium-low micromolar range were needed for a 50% relaxation [34,35]. An extract of the prenylflavone-containing hops was reported to exert relaxant activity on rat ileum contractions ex vivo, but since the extracting solvent was water, it is unlikely that the active constituents were prenylated phenolics [36]. 

Despite the widely available options for antispasmodic therapy, there has been an increase in complaints related to bowel motility and bronchial asthmatic problems [37,38]. Therefore, there is an emerging need to find new, effective and safe, possibly natural drugs for the treatment of such disorders. The potent antispasmodic compounds presented in this work may open the way towards potential new therapeutic alternatives to the existing treatments. 

## 3. Materials and Methods 

### 3.1. General

Column chromatography (CC), flash chromatography (FC), and thin layer chromatography (TLC) were performed on polyamide SC6 (50–160 µm, Macherey-Nagel GmbH and Co., Düren, Germany), silica gel 60 (45–63 µm, Molar Chemicals, Halásztelek, Hungary), and silica gel 60 F254 or RP-18 F254S (250 µm, Merck Co., Darmstadt, Germany), respectively. TLC was used at each chromatographic step to monitor the separation with a solvent system of toluene–ethyl acetate–formic acid (5:4:1, *v/v/v*), and the spots were detected under UV light (λ1 = 254 nm, λ2 = 365 nm) and daylight after spraying with 5% H_2_SO_4_ in ethanol followed by heating. HPLC analysis was performed on a Jasco 2010 series instrument equipped with a Jasco PU-2080 quaternary pump, a vacuum degasser, an AS-2055Plus intelligent autosampler and a Jasco MD-2010 Plus photodiode array detector (Jasco Co., Tokyo, Japan). Melting points were determined on a Boetius apparatus (VEB Analytik Dresden, Dresden, Germany). Organic solvents used for TLC, FC and CC (analytical grade) were purchased from Sigma-Aldrich (Budapest, Hungary), and HPLC solvents were purchased from Avantor Performance Materials (Gliwice, Poland). Papaverine was purchased from Takeda Pharma Ltd. (Budapest, Hungary).

### 3.2. Plant Material, Extraction and Pre-Purification 

Root bark (590 g) of *Morus nigra* was collected from a farm near Ásotthalom, Hungary, in December 2013. After air-drying, a voucher specimen was deposited in the Institute of Pharmacognosy, University of Szeged, Szeged, Hungary. The plant material was ground into a coarse powder and extracted with MeOH (3 × 4 L) at room temperature. After evaporation of methanol under vacuum at 40 °C, the residue (92.7 g) was diluted with 18% of MeOH. Following this, the crude extract was subjected to solvent-solvent distribution, first with *n*-hexane (5 × 500 mL), and then with ethyl acetate (5 × 500 mL) to produce *n*-hexane (7.3 g), ethyl acetate (54.1 g) and water (36.7 g) fractions. Ethyl acetate fraction was further processed due to its higher amount and chemical complexity.

### 3.3. Chromatographic Separation of Compounds ***1***–***7***

Column chromatographic separation of compounds was performed in a glass column (162 cm × 5.5 cm). The column was packed with 500 g polyamide and the ethyl acetate layer of the crude extract was subjected to the column through dry loading technique by adsorbing it on polyamide (100 g). A stepwise gradient of ethyl acetate and mixtures of ethyl acetate–methanol (95:5, 9:1, 8:2 and 7:3, *v/v*) was utilized for the separation. The eluates were evaporated under vacuum to obtain 258 fractions, and fractions were joined according to their TLC fingerprints to afford a total of 56 joint fractions that were subsequently further purified by flash chromatography and HPLC.

Flash chromatography was performed for fractions 7, 13, 20 and 25 on a CombiFlash Rf+ Lumen instrument equipped with an integrated evaporative light scattering detector (ELSD) (Teledyne Isco, Lincoln, NE, USA). Fraction 7 (10 g) was purified on a silica gel column (80 g) gradually eluted with *n*-hexane–ethyl acetate to afford 24 subfractions giving pure compound **1** (1.98 g) from the subfraction 10, and the subfraction 17 was purified by preparative HPLC (Waters Co. Milford, MA, USA), using ACN–H_2_O (7∶3, *v/v*) as a mobile phase, leading to the isolation of compound **2** (85 mg). The column fraction 13 (1.27 g) underwent reverse phase chromatography over polyamide column (13 g polyamide) by using dry loading on cellite (20–100 µm), with a stepwise gradient of aqueous MeOH as an eluting agent through flash in order to obtain nine subfractions affording pure compound **3** (60 mg) from the 6th flash fraction, whereas subfraction 8 was purified over RP-HPLC on a C18 (5 µm, 250 × 21.2 mm) column with an isocratic elution of ACN–H_2_O (1:1, *v/v*). The purified fraction (75 mg) was further separated on a Biphenyl (5 µm, 250 × 21.2 mm) column with the help of preparative HPLC using MeOH–H_2_O (57:43, *v/v*) to acquire compounds **4** (30 mg) and **5** (35 mg). Fraction 20 (1.25 g) was purified over polyamide through reverse-phase flash chromatography with aqueous MeOH as the mobile phase, and subfraction 5 was further purified on a C18 column using ACN–H_2_O (5∶5, *v/v*) to get compound **6** (120 mg). Fraction 25 (1.21 g) was gradually eluted with aqueous MeOH to obtain compound **7** (180 mg) through reverse phase flash chromatography using polyamide column. After the solvent was evaporated, the purified compounds were stored in a refrigerator until further analysis.

The relative purity of each isolated compound was examined by HPLC using a C18 column with a flow rate of 1.0 mL/min, with an isocratic elution of ACN–H_2_O (1:1, *v/v*), and 20 µL of each sample was injected. 

### 3.4. Structure Elucidation of the Isolated Compounds

Structure determination of the isolated compounds was carried out by one- (^1^H, ^13^C, APT) and two-dimensional (HSQC, HMBC, ^1^H,^1^H-COSY, NOESY) NMR spectroscopic methods. NMR spectra were recorded at room temperature in methanol-*d*_4_ on Bruker Avance DRX 500 and NEO spectrometers, however, for compound **5**, the 600/150 MHz spectra were taken in dimethylsulfoxide-*d*_6_ on a Bruker Avance III 600 MHz spectrometer equipped with Prodigy cryo-probe head. Chemical shifts (*δ*) are given on the δ-scale and referenced to the solvents (methanol-*d*_4_: δ_H_ = 3.31 and δ_C_ = 49.1 ppm, and dimethylsulfoxide-*d*_6_: δ_H_ = 2.50 and δ_C_ = 39.5 ppm) and coupling constant (*J*) values are expressed in Hz. Pulse programs of all experiments [^1^H, ^13^C, APT, gs-HSQC, edited gs-HSQC, gs-HMBC (optimized for 7 Hz), band-selective gs-HMBC and NOESY (mixing time = 300 ms)] were taken from the Bruker software library. The NMR signals of the products were assigned by comprehensive one- and two-dimensional NMR methods using widely accepted strategies [39,40]. Most ^1^H assignments were accomplished using general knowledge of chemical shift dispersion with the aid of the proton–proton coupling pattern (^1^H-NMR spectra). High-resolution mass spectra (HRESIMS) were recorded on a Thermo Scientific Q Exactive Plus orbitrap mass spectrometer equipped with a HESI-II ion source (Waltham, MA, USA). Samples were applied by using flow injection by a Thermo Dionex Ultimate 3000 HPLC system. The eluent was water–acetonitrile (1:1, *v/v*) mixture containing 0.1% formic acid, the flow rate was 200 µL/min, and 5 µL of the sample was injected. The ion source parameters were set as follows: nebulizer gas flow: 10, auxiliary gas: 18, sheath gas: 3, all in arbitrary units, the auxiliary gas temperature was 400 °C, and the ion transfer capillary temperature was 300 °C. Theoretical *m/z* values for the [M + H]^+^ molecular ions were calculated by ChemDraw 12.0 (CambridgeSoft, Cambridge, MA, USA). HRESIMS spectra of compounds **1**–**5** are provided as Supplementary Materials, Figures S15-S19.

*Morusin* (**1**): yellow solid [23]; ^1^H-NMR (CD_3_OD, 500 MHz) *δ* 7.10 (1H, d, *J* = 8.2 Hz), 6.58 (1H, d, *J* = 10.0 Hz), 6.42 (1H, br s), 6.40 (1H, br d, *J* = 8.2 Hz), 6.14 (1H, s), 5.57 (1H, d, *J* = 10.0 Hz), 5.09 (1H, br t, *J* = 6.9 Hz), 3.10 (2H, d, *J* = 7.3 Hz), 1.59 (3H, s), 1.43 (6H, s), 1.40 (3H, s); ^13^C-NMR (CD_3_OD, 125 MHz) *δ* 183.9, 163.6, 162.7, 162.1, 160.5, 158.0, 153.8, 132.9, 132.5, 128.2, 122.7, 122.1, 115.8, 113.1, 108.0, 105.9, 103.8, 102.2, 100.1, 79.1, 2 × 28.4, 25.9, 24.9, 17.7. HRESIMS: C_25_H_24_O_6_ ([M + H]^+^ calcd. *m/z*: 421.16511, found *m/z*: 421.16407).

*Kuwanon U* (**2**): yellow solid [26]; ^1^H-NMR (CD_3_OD, 500 MHz) *δ* 7.13 (1H, s), 6.43 (1H, s), 5.91 (1H, br s), 5.88 (1H, br s), 5.62 (1H, br d, *J* = 12.3 Hz), 5.24 (1H, br t, *J* = 7.0 Hz), 5.08 (1H, br t, *J* = 6.0 Hz), 3.78 (3H, s), 3.19 (2H, d, *J* = 7.2 Hz), 3.02 (1H, dd, *J* = 17.0 and 13.5 Hz), 2.70 (1H, br d, *J* = 17.0 Hz), 2.08 (2H, m), 2.01 (2H, m), 1.67 (3H, s), 1.61 (3H, s), 1.55 (3H, s). ^13^C-NMR (CD_3_OD, 125 MHz) *δ* 198.3, 168.3, 165.5, 165.3, 159.5, 154.7, 136.4, 132.1, 128.4, 125.4, 124.4, 122.1, 117.8, 103.4, 99.6, 96.9, 96.2, 75.9, 55.8, 43.1, 40.8, 28.6, 27.8, 25.9, 17.8, 16.2. HRESIMS: C_26_H_30_O_6_ ([M + H]^+^ calcd. *m/z*: 439.2121, found *m/z*: 439.2112). 

*Kuwanon E* (**3**): yellow solid [24]; ^1^H-NMR (CD_3_OD, 500 MHz) *δ* 7.07 (1H, s), 6.34 (1H, s), 5.91 (1H, br s), 5.88 (1H, br s), 5.62 (1H, br d, *J* = 12.3 Hz), 5.30 (1H, br t, *J* = 6.6 Hz), 5.11 (1H, m), 3.20 (2H, d, *J* = 7.1 Hz), 3.05 (1H, dd, *J* = 17.0 and 12.8 Hz), 2.70 (1H, br d, *J* = 17.0 Hz), 2.09 (2H, m), 2.02 (2H, m), 1.68 (3H, s), 1.62 (3H, s), 1.56 (3H, s); ^13^C-NMR (CD_3_OD, 125 MHz) *δ* 198.5, 168.3, 165.5, 165.4, 157.0, 154.4, 136.5, 132.1, 128.7, 125.4, 124.4, 120.6, 117.3, 2 x 103.4, 96.9, 96.2, 76.1, 43.2, 40.9, 28.5, 27.8, 25.9, 17.8, 16.2. HRESIMS: C_25_H_28_O_6_ ([M + H]^+^ calcd. *m/z*: 425.16941, found *m/z*: 425.19619).

Contradictory data have been published concerning the NMR chemical shifts of moracins P, R and O, and this may lead to a mistaken identification of these. Therefore, we present all relevant NMR spectra for compounds **4** and **5** as Supplementary Material, Figures S1-S14.

*Moracin P* (**4**): brown solid [25]; m.p. 252–254 °C; ^1^H-NMR (CD_3_OD, 500 MHz) *δ* 7.23 (1H, s, H-4), 6.89 (1H, s, H-3), 6.86 (1H, s, H-7), 6.75 (2H, d, *J* = 2.1 Hz, H-2ʹ,6ʹ), 6.24 (1H, br t, *J* = 2.0 Hz, H-4ʹ), 3.79 (1H, dd, *J* = 7.6 and 5.2 Hz, H-2″), 3.12 (1H, dd, *J* = 16.5 and 5.2 Hz H-1″a), 2.83 (1H, dd, *J* = 16.5 and 7.6 Hz H-1″b), 1.36 (3H, s, H-5″), 1.28 (3H, s, H-4″); ^13^C-NMR (CD_3_OD, 125 MHz) *δ* 160.1 (C-3ʹ,5ʹ), 156.7 (C-2), 156.0 (C-7a), 152.7 (C-6), 133.8 (C-1ʹ), 124.3 (C-3a), 121.9 (C-4), 117.8 (C-5), 104.1 (C-2ʹ,6ʹ), 103.7 (C-4ʹ), 101.9 (C-3), 99.8 (C-7), 78.3 (C-3″), 70.7 (C-2″), 32.5 (C-1″), 26.1 (C-5″), 21.2 (C-3″). HRESIMS: C_19_H_18_O_5_ ([M + H]^+^ calcd. *m/z*: 327.1232, found *m/z*: 327.1230).

*Moracin O* (**5**): brown solid [25]; m.p. 235–236 °C; ^1^H-NMR (CD_3_OD, 500 MHz) *δ* 7.29 (1H, s, H-4), 6.89 (1H, s, H-3), 6.85 (1H, s, H-7), 6.74 (2H, d, *J* = 2.1 Hz, H-2ʹ,6ʹ), 6.23 (1H, br t, *J* = 2.1 Hz, H-4ʹ), 4.64 (1H, t, *J* = 8.6 Hz, H-2″), 3.22 (2H, m, H_2_-1″), 1.28 (3H, s, H-4″), 1.24 (3H, s, H-5″); ^13^C-NMR (CD_3_OD, 125 MHz) *δ* 160.1 (C-3ʹ,5ʹ), 160.0 (C-6), 156.5 (C-7a), 156.3 (C-2), 133.9 (C-1ʹ), 125.2 (C-5), 124.1 (C-3a), 117.1 (C-4), 103.9 (C-2ʹ,6ʹ), 103.5 (C-4ʹ), 102.5 (C-3), 93.3 (C-7), 91.5 (C-2″), 72.6 (C-3″), 31.3 (C-1″), 25.4 (C-4″), 25.5 (C-5″). ^1^H-NMR (DMSO-D_6_, 600 MHz) *δ* 9.43 (2H, s, HO-3ʹ,5ʹ), 7.34 (1H, s, H-4), 7.07 (1H, s, H-3), 6.97 (1H, s, H-7), 6.66 (2H, d, *J* = 2.1 Hz, H-2ʹ,6ʹ), 6.19 (1H, t, *J* = 2.1 Hz, H-4ʹ), 4.63 (1H, s, HO-3”), 4.60 (1H, dd, *J* = 9.3 and 8.3 Hz, H-2″), 3.20 (1H, dd, *J* = 15.7 and 8.3 Hz, H_a_-1″), 3.15 (1H, dd, *J* = 15.7 and 9.3 Hz, H_b_-1″),1.144 (3H, s, H-5″), 1.137 (3H, s, H-4″); ^13^C-NMR (DMSO-D_6_, 150 MHz) *δ* 158.8 (C-3ʹ,5ʹ), 158.3 (C-6), 154.2 (C-7a), 154.1 (C-2), 131.7 (C-1ʹ), 124.2 (C-5), 121.8 (C-3a), 116.1 (C-4), 102.5 (C-4ʹ), 102.1 (C-2ʹ,6ʹ), 101.7 (C-3), 92.2 (C-7), 90.0 (C-2″), 70.1 (C-3″), 29.5 (C-1″), 26.1 (C-4″), 24.8 (C-5″). HRESIMS: C_19_H_18_O_5_ ([M + H]^+^ calcd. *m/z*: 327.1232, found *m/z*: 327.1227).

*Albanol A* (**6**): yellow solid [27]; ^1^H-NMR (CD_3_OD, 500 MHz) *δ* 7.33 (1H, d, *J* = 8.4 Hz), 7.12 (1H, d, *J* = 8.7 Hz), 7.08 d (1H, d, *J* = 8.4 Hz), 6.91 (1H, s), 6.89 (1H, br s), 6.88 (1H, br s), 6.81 (1H, br s), 6.72 (1H, dd, *J* = 8.4 and 1.3 Hz), 6.44 (1H, dd, *J* = 8.4 and 1.8 Hz), 6.40 (1H, br s), 6.33 (1H, d, *J* = 1.8 Hz), 6.31 (1H, d, *J* = 1.7 Hz), 6.13 (1H, dd, *J* = 8.7 and 1.7 Hz), 3.33 (1H, overlapped with w signal), 3.31 (1H, overlapped with solvent signal), 2.94 (1H, ddd, *J* = 11.5 and 11.0 and 5.0 Hz), 2.66 (1H, dd, *J* = 16.9 and 5.0 Hz), 2.00 (1H, dd, *J* = 16.9 and 11.0 Hz), 1.78 s (3H, s); ^13^C-NMR (CD_3_OD, 125 MHz) *δ* 160.1, 158.4, 157.9, 157.8, 157.3, 156.8, 155.7, 154.9, 153.6, 133.9, 131.5, 130.6, 128.0, 123.4, 123.1, 122.0, 118.3, 117.4, 113.9, 113.2, 110.0, 107.0, 105.5, 105.0, 104.5, 104.2, 103.1, 102.1, 98.5, 37.6, 36.7, 35.4, 28.8, 23.9.

*Albanol B* (**7**): dark brown solid [27]; ^1^H-NMR (CD_3_OD, 500 MHz) *δ* 8.38 (1H, s), 7.59 (1H, d, *J* = 8.3 Hz), 7.50 (1H, s), 7.33 (1H, d, *J* = 8.3 Hz), 7.01 (1H, br s), 6.99 (1H, br s), 6.95 (1H, s), 6.90 (1H, br s), 6.73 (1H, dd, *J* = 8.3 and 1.9 Hz), 6.51 (1H, dd, *J* = 8.3 and 1.8 Hz), 6.47 (1H, br s), 6.26 (1H, d, *J* = 2.1 Hz), 6.13 (1H, d, *J* = 8.6 Hz), 5.85 (1H, dd, *J* = 8.6 and 2.1 Hz), 2.50 (3H, s); ^13^C-NMR (CD_3_OD, 125 MHz) *δ* 160.6, 160.0, 158.1, 2 × 157.3, 156.9, 155.0, 153.5, 152.6, 140.9, 132.6, 131.5, 130.0, 129.7, 126.6, 125.3, 123.0, 122.5, 122.3, 121.3, 115.9, 115.6, 113.4, 111.7, 111.5, 107.3, 106.6, 106.4, 106.0, 105.4, 104.9, 103.2, 98.5, 22.3.

### 3.5. Housing and Handling of the Animals

All experiments involving animal subjects were carried out with the approval of the National Scientific Ethical Committee on Animal Experimentation (permission number: IV/198/2013). The animals were treated in accordance with the European Communities Council Directives (2010/63/EU) and the Hungarian Act for the Protection of Animals in Research (Article 32 of Act XXVIII).

Sprague-Dawley rats were maintained at 22 ± 3 °C, under a lights-darkness cycle of 12 h/12 h, with a relative humidity of 30%–70%. The animals were fed with a standard rodent pellet diet (Innovo Ltd., Isaszeg, Hungary), with tap water available ad libitum.

### 3.6. Isolated Organ Bath Studies

In isolated organ bath studies, male rats (160–200 g, *n* = 8) were used. The animals were starved 16 h before the experiment. On the day of measurement, rats were terminated by CO_2_ inhalation. 

Distal ileum was dissected, cut into 5 mm long samples, and mounted vertically in an organ bath containing 10 mL of Tyrode buffer (composition in mM: 137 NaCl, 3 KCl, 1 CaCl_2_, 1 MgCl_2_, 12 NaHCO_3_, 0.4 NaH_2_PO_4_, 6 glucose, pH 7.4), heated at 37 °C and carbogen (95% O_2_ + 5% CO_2_) was bubbled through it. The initial tension of the ileum samples was set to about 1.5 g, the tissues were equilibrated for about 60 min, and the buffer was changed in every 15 min. The regular contractions were recorded for 5 min, and then 25 mM KCl was administered. The area under the curve (AUC) of KCl evoked contractions was compared with the AUC of regular contractions. Papaverine was used as a positive control. 

After the esophagus and blood vessels were removed from trachea tissues, they were cut transversally into 4–5 mm-wide rings, which were placed in 37 °C Krebs buffer (composition in mM: 118 NaCl; 4.75 KCl; 2.5 CaCl_2_; 1.19 K_2_HPO_4_; 25 NaHCO_3_; 1.2 MgSO_4_ and 11 glucose, pH 7.4). The tracheal rings were mounted with their longitudinal axis vertically by hooks and equilibrated for 1 h, while the buffer solution was renewed in every 15 min. The initial strain was set to about 2.00 g. Papaverine was used as a positive control in the same concentration range as that of the isolated compounds.

First, we carried out a 3-points assay (10^−8^, 10^−7^ and 10^−6^ M) to determine the approximate activities of the compounds, then the compounds showing significant activity (i.e., maximum ileum contraction inhibition higher than 30%, and tracheal tone reduction higher than 100 mg) were studied further in an 8-points assay (10^−8.5^–10^−5^ M) obtaining cumulative concentration-response curves. The concentrations eliciting the half of the maximum effect (EC_50_) and the maximum effects (E_max_) were calculated and statistically evaluated.

The tracheal and ileal tissues activities were measured with a gauge transducer (SG-02, MDE Ltd, Budapest, Hungary) and recorded with a SPEL Advanced ISOSYS Data Acquisition System (MDE Ltd, Budapest, Hungary).

### 3.7. Statistical Analysis

All smooth muscle contractility data were analyzed by the Prism 5.01 (GraphPad Software, USA) software. The data were statistically evaluated by ANOVA with a Tukey multiple comparison test that is suitable to compare more than two groups. The Tukey multiple comparison test compares every mean with every other mean, and for each dataset, we present statistically significant differences as compared with the papaverine group.

## 4. Conclusions

Seven phenolic compounds were isolated from the root bark of *Morus nigra*. The structure elucidation of compounds **4** and **5** revealed contradictory literature data that allow misidentification and confusing the identities of moracin derivatives. Through a detailed NMR investigation, we have unambiguously identified compound **4** as moracin P and compound **5** as moracin O, and provided their complete NMR signal assignment in DMSO-*d_6_* and/or CD_3_OD. 

Subsequent to an ex vivo antispasmodic screening on rat ileal and tracheal smooth muscles, three compounds were selected for a detailed investigation. Based on our results, compounds **2**, **5** and **7** were identified as potential new antispasmodic agents. The affinity and efficacy of kuwanon U (**2**) and albanol B (**7**) make these compounds equipotent with papaverine, and this would warrant their further studies in intact animals. Most importantly, moracin O (**5**) was found superior to papaverine in both smooth muscle organs. Accordingly, this compound was identified as a new lead molecule that may be further developed to a potent new gastrointestinal or tracheal relaxant, and/or may serve as a chemical starting point towards the development of a new class of spasmolytic drugs.

## Figures and Tables

**Table 1:** Activity of the crude extract, pre-purified fractions and purified constituents of *Morus nigra* L. in a 3-point assay on isolated rat ileum and trachea. Papaverine was used as a positive control. The extract and fractions were administered at 0.01, 0.03 and 0.1 mg/mL, and the pure compounds at 10^−8^, 10^−7^ and 10^−6^ M concentrations.

Compound	Maximal Inhibition of Ileal Contraction (%)	Tracheal Tone Reduction (mg)
papaverine ^a^	47.9 ± 10.5	134.4 ± 41.4
MeOH extract	−33.2 ± 27.9 ***	−22.0 ± 11.9 ***
EtOAc fraction	24.4 ± 16.2 **	−5.0 ± 11.9 ***
*n*-Hexane fraction	no action	no action
Water fraction	−9.1 ± 10.8 ***	−32.5 ± 10.3 ***
morusin (**1**)	no action	90.7 ± 20.4 *
kuwanon U (**2**) ^a^	39.6 ± 4.5	145.5 ± 16.7
kuwanon E (**3**)	26.6 ± 9.1 *	no action
moracin P (**4**)	no action	42.4 ± 7.7 **
moracin O (**5**) ^a^	47.2 ± 11.6	123.6 ± 36.1
albanol A (**6**)	20.5 ± 9.1 **	194.7 ± 42.3 *
albanol B (**7**) ^a^	35.5 ± 8.4	100.6 ± 43.6 *

^a^ Compounds involved in further experiments due to their significant activity on both types of smooth muscle (criterion set as >30% inhibition of ileal contraction and >100 mg reduction in the tracheal tone). The negative actions mean contracting effect. Papaverine was used as a positive control on both experimental models. *p < 0.05, **p < 0.01 and ***p < 0.001 as compared to the effect of papaverine by means of one-way ANOVA followed by Tukey’s post-hoc test.

**Table 2:** Smooth muscle relaxant activity of compounds **2**, **5** and **7** on isolated rat ileum and trachea. EC_50_ and E_max_ values on the ileal contractions and tracheal tone are presented. Papaverine was used as a positive control on both experimental models.

Compound	Ileal Contractions		Tracheal Tone	
	EC_50_ ± SEM (µM)	E_max_ ± SEM (%)	EC_50_ ± SEM (µM)	E_max_ (mg ± SEM)
kuwanon U (**2**)	0.13 ± 0.04	70.5 ± 6.1	0.033 ± 0.05	247.8 ± 9.9
moracin O (**5**)	1.1 ± 0.43	85.3 ± 4.4*	0.062 ± 0.01	309.5 ± 17.7 *
albanol B (**7**)	1.3 ± 0.98	83.2 ± 3.9	0.100 ± 0.05	254.9 ± 19.3
papaverine	0.44 ± 0.15	63.6 ± 6.3	0.074 ± 0.03	233.7 ± 15.4

*p < 0.05 as compared to the effect of papaverine by means of one-way ANOVA followed by Tukey’s post-hoc test.

## Supplementary Materials

NMR spectra of compounds **4** and **5**, and HRESIMS spectra of compounds **1**–**5** are provided as supplementary material. These can be found at https://www.mdpi.com/1420-3049/24/13/2497/s1, Figures S1–S19.
